# Microencapsulation Techniques in HIV Pediatric Formulations: Advances and Future Outlook

**DOI:** 10.1155/2024/5081655

**Published:** 2024-10-09

**Authors:** Nnamdi Ikemefuna Okafor

**Affiliations:** ^1^Department of Pharmaceutical Sciences, University of the Western Cape, Robert Sobukwe Drive, Bellville, Cape Town, South Africa; ^2^Wits Advanced Drug Delivery Platform Research Unit, Department of Pharmacy and Pharmacology, School of Therapeutic Sciences, Faculty of Health Sciences, University of Witwatersrand, Johannesburg, South Africa

## Abstract

The treatment of human immunodeficiency virus (HIV) in children has persistently been complex and tedious on a global scale. This is because adult and pediatric HIV treatments follow a similar therapeutic approach. Due to the dearth of clinically licensed pediatric antiretroviral drug (ARVD) therapy, children with HIV worldwide are prescribed unlicensed drugs each year. This has triggered likelihood of poor drug adherence, therapeutic failure, and even adverse reactions brought on by a variety of factors, including pill size and quantity, which is the main cause of swallowing difficulties, repeated administration of these various ARVDs, many of which have poor solubility and cause severe side effects in children, and unpalatability of the drug, which is one of the criteria for pediatric formulations. Thus, there is a necessity for investigation into several advanced microencapsulation techniques that could curb these challenges. Microencapsulation techniques have explored in drug delivery for encapsulation and manufacture of different nanoparticles that have shown significant potential in mitigating and surmounting different constraints, such as taste masking, enhanced drug solubility and bioavailability, and production of micronized fine powders for treatment of varying diseases. Nevertheless, the usage of these technologies in HIV pediatric formulations has garnered relatively little attention. Thus, this review has paid a keen interest in examining several microencapsulation strategies for potential utilization in the development of HIV pediatric formulations.

## 1. Introduction

**Figure 1:**
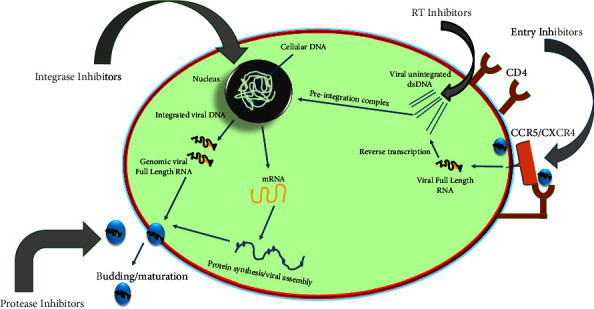
HIV life cycle diagram with antiretroviral treatment targets adapted from [8].

The acquired immunodeficiency syndrome (AIDS) caused by the human immunodeficiency virus (HIV) is regarded as the second most prevalent cause of mortality for adolescents globally especially in the underdeveloped nations [1, 2]. In accordance with an updated report by the Joint United Nations Programme on HIV/AIDS (UNAIDS), the sub-Saharan Africa is the most severely afflicted, with approximately 37.9 million people worldwide living with this disease, encompassing 2.1 million children under the age of 15 years [3]. Notwithstanding persistent efforts to avert vertical HIV transmission, about 1500 new cases of pediatric HIV infection are reported every day and 15% of new HIV infections are reported each year in children. This has resulted to children accounting for 18% of HIV-related mortality worldwide, and as a result, without antiretroviral therapy (ART), half of children diagnosed with HIV die before the age of two while 80% die by the age of five [4, 5]. The use of ART has demonstrated high therapeutic efficacy in treating pediatric HIV disease in both low- and high-income countries; however, only 58% of the 2.1 million children living with HIV were found to be receiving treatment. The ART treatment strategy was primarily developed for the management of the viral infection and controlling of the disease progression, thereby improving the quality of life of the patient [6]. This is by improving the prognosis of HIV-related individuals, reducing HIV-related mortality and morbidity while minimizing other opportunistic infections [5, 7]. Due to the long-lasting viral suppression and resulting decrease in morbidity and death, ART is regarded as the best therapy which has led to the recommendation of over 23 antiretroviral drugs (ARVDs) by the US Food and Drug Administration (FDA), as shown in Table 1. Two nucleoside reverse transcriptase inhibitors (NRTIs), which serve as the foundation of standard ART regimens for both adults and children, are combined with any other third ARV agents, such as a non-nucleoside reverse transcriptase inhibitor (NNRTI), a protease inhibitor (PI), or an integrase inhibitor (II). Since ART targets the viral transcriptase or the protease (Figure 1), it suppresses the plasma viral load and usually results in increased CD4 counts within 3 to 6 months with improved clinical staging despite the fact that current medications do not completely eradicate HIV-1 infection [9–12]. Despite the effectiveness of the ARVDs, mostly among adult patients, poor adherence has been linked to the development of drug resistance and treatment failure, making it a critical concern for HIV patients on ART, specifically with children receiving the medication. Only around half of the children who require ART could get or afford them, despite the huge rise in HIV patients receiving ART—from 3% in 2000 to 46% in 2015. The sad fact that only 6% (35 of 583) of clinical ARVDs were pediatric formulation with only 5% of all subjects shows that children are not prioritized enough in the development of appropriate ARVDs, in contrast to adults, despite the fact that children make up 4.9% of the world's HIV population [13]. Only 58% of the 2.1 million children with HIV who are currently alive are being treated, and most of these children come from developing nations and cannot afford ART. By enhancing the prognosis of HIV-related individuals, lowering HIV-related mortality and morbidity, and minimizing other opportunistic infections, the use of ARVDs has demonstrated high therapeutic efficacy in the treatment of HIV disease, particularly among adults in both low- and high-income countries [5, 7]. However, this is not the case in children given the stigma associated with HIV, the cost of medications, the difficulty of swallowing and dose adjustment, the dispensability of and complexity of regimens, the challenge of bitter taste, and drug resistance due to long-term therapy which are just a few of the issues that prevent children them from maintaining treatment adherence. These negative aspects have also contributed to a number of serious adverse reactions in children, including hypersensitive reaction, lactic acidosis, hyperlipidaemia, and bone demineralization, which are incompatible with adults taking ART [10, 13]. In this context, it is urgently necessary to enhance present ARV dosage forms and make them more children friendly. And cognizant of these constraints, this work has examined prospective microencapsulation techniques that could be utilized in developing innovative pediatric formulations that can minimize these limitations especially with the solubility and concealing the unpalatable taste of licensed ARVDs for efficient HIV pediatric therapy [14–17].

## 2. Microencapsulation

**Figure 2:**
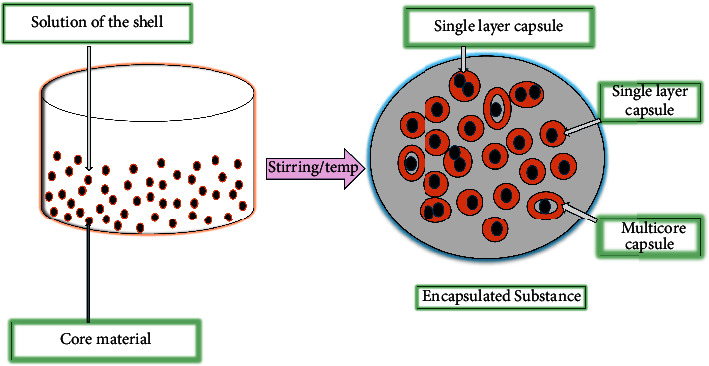
An abridged diagram illustrating the microencapsulation process within nanocarrier adapted from [18].

Microencapsulation (Figure 2) is the science or technology used to encapsulate a solid or liquid core substance by trapping this material by a wall forming or carrier. Typically, this procedure yields tiny solid particles ranging in dimension from nanometres to micrometres [19, 20]. Schleicher and Green developed and patented the notion of microencapsulation technology in the 1950s for the fabrication of dye-encapsulated capsules designed just for paper copying [21, 22]. Since then, microencapsulation has been used and researched in a variety of disciplines and industries, including agriculture, electronics, pharmaceuticals, food, cosmetics, and biomedical [23]. Microencapsulation has shown several advantages, particularly in the agricultural, food, and pharmaceutical industries, and has given their interesting features which consist of the encapsulation of adhesives, dyes, live cells, active enzymes, flavors, fragrances, drugs, their controlled or delayed release of active ingredients, masking of unpleasant taste, and enhanced solubility [24, 25].

**Figure 3:**
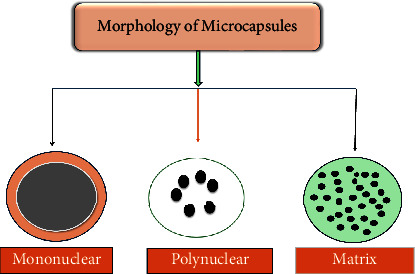
Classification of various forms of microcapsule as adapted from [26].

The microencapsulation technique is known for producing microparticles which can be subdivided into microspheres or microcapsules. These microcapsules vary in size and shape according to the procedure and materials used through the addition of multiple polymers or monomers through diverse microencapsulation procedures [22]. These microcapsules can be divided into three categories, as shown in Figure 3, mononuclear (the core is encased by the shell), polynuclear (multiple core materials are enclosed within the shell), and matrix (the cores are equally or uniformly spread throughout the shell).

### 2.1. Applications of Microencapsulation

**Figure 4:**
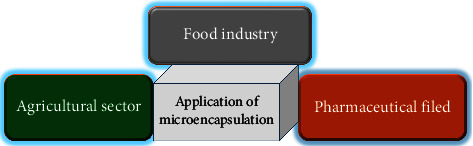
Application of microencapsulation in different fields.

The application of microencapsulation has gained traction in a variety of industries; it has piqued the interest of researchers and product creators, and as a result, it has been used in a variety of fields including but mostly in fabricating drug carrier or delivery agent in pharmaceutical and biomedical fields (Figure 4). Although other applications of microencapsulation have been highlighted, the major emphasis of this work has been on its use in pharmaceutical industries for drug delivery.

#### 2.1.1. Application in Food Industry

Microencapsulation in the food business has gained substantial attention [20, 27, 28]. Some food-active chemicals degrade rapidly when exposed to oxygen, light, certain pH levels, and food processing conditions [29]. As a result, microencapsulation in the food business strives to reduce food ingredient reactivity to environmental and processing conditions [21]. Because several active compounds are volatile in nature, this technology is required to reduce the growing concern of waste and evaporation of volatile ingredients to the processing medium, as well as masking of unpleasant flavors from active ingredients and allowing homogeneous distribution of food ingredients [29]. This technology has greatly improved the application of microencapsulation in the food industry, as well as the addition of beneficial substances [21].

#### 2.1.2. Application in the Agricultural Sector

The attack of insects on agricultural produce has focused attention on effective strategies to avoid crop loss [27, 28]. The use of synthetic pheromones has emerged as a viable alternative to traditional pest management methods in agriculture. Sex pheromone is viewed as a tool for bulk insect capture, population inspection, and disrupting the mating process between male and female insects [20]. In this context, microencapsulation has been used in the production of encapsulated insect attractant pheromones employing various polymers as microencapsulants such as polyurea, Arabic gum, and gelatin [20, 30]. Microencapsulation has also been used in agriculture to reduce the chemical impact of fertilizers on crop output quality [31]. Previous research studies on encapsulated fertilizer in a polymeric microstructure of urea explored the biological activity on target plants maize and sunflower [31].

#### 2.1.3. Application in the Pharmaceutical Industry

In the pharmaceutical industry, the common goal of microencapsulation is to discover the most effective drug delivery system (DDS) that will be widely accepted for the treatment of a specific disease, thereby reducing side effects and reactions from the drugs, targeting the specific site of action, promoting sustained or prolonged release of the compound, increasing shelf lives, and improving patient compliance [21, 32]. Numerous active pharmaceutical ingredients (APIs) have been reported to have short half-lives, which could be addressed by using microparticles with prolonged drug release. Many medications have poor chemical stability and breakdown easily through hydrolysis or oxidation. Furthermore, many APIs are recognized for their metallic, salty, or bitter taste, making them disagreeable to both children and adults, resulting in poor adherence among patients, particularly in cases of repeated administration [33, 34]. API microencapsulation is thus an approach aimed at addressing some or all the difficulties. This pharmaceutical preformulation technology is not new to the pharmaceutical business, as seen in the following list of approved and sold microencapsulated drugs (Table 2).

### 2.2. Microencapsulation Techniques

Numerous microencapsulation techniques have been investigated in the encapsulation of the desired microcapsules considering the size and shape, as well as the physicochemical properties of the core material and the encapsulating agents (Table 3) [69]. Each technique has different features, identifications, adaptations, and specifications. Many of these techniques have been explored in the encapsulation of ARVDs which have given rise to licensed and approved HIV drug today majorly for adults. This is because these techniques can improve drug bioavailability while overcoming issues with poor solubility, poor palatability, and slow dissolution rates. They are also capable of producing particles with appropriate aerodynamic diameters and particles within a certain size range [69]. This is due to the fact that, according to the article, particle size affects solubility and dissolution rates because micronization or particle size reduction increases the surface area on which water interacts, which increases the rate of dissolution and bioavailability of active ingredients, especially for compounds with low water solubility [69, 70]. Despite their huge potentials towards HIV adult formulations, little or no attention has been given to HIV pediatric formulation using these techniques. Thus, some of these techniques and their distinctive properties have been assessed towards HIV pediatric formulations to help minimize the challenges associated with the treatment of children living with HIV. This is because their accomplishments have been confirmed as they have shown promise in tackling challenges associated with the use of ARVDs.

#### 2.2.1. Spray Drying

**Figure 5:**
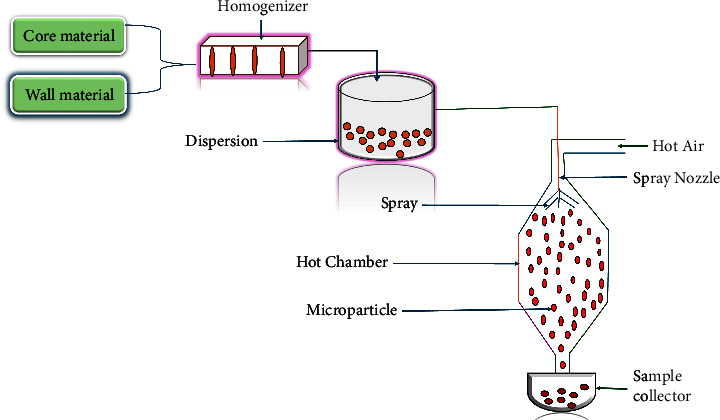
Diagrammatic representation of spray-drying technique for microencapsulation adapted from [71].

One of the extensively and often used microencapsulation procedures for different active components is spray drying (Figure 5). This technique involves dissolving the active substance or chemical in a polymer solution to create a dispersed polymer suspension, which is then sprayed into a heated chamber. It involves intricate interactions between process, equipment, and feed factors, all of which have an impact on the quality of the finished product [72]. During the functioning of a spray drying machine, a liquid product is atomized in a hot gas current to rapidly generate a powder. The gas that is most usually utilized is air, or less frequently, an inert gas, typically nitrogen gas. The initial liquid feeding can be a solution, emulsion, or suspension and can be used on both heat-sensitive and heat-resistant items. Microcapsules of polynuclear and matrix form as a result of the coating material solidifying onto the active particles during the solvent-induced evaporation [19, 22, 72]. The final product's physicochemical characteristics are primarily influenced by the input temperature, air flow rate, feed flow rate, atomizer speed, and the kinds and concentrations of carrier agents [72]. Since being discovered in 1937 by Boake Roberts, this method has been the most widely and often used encapsulating method. Since its invention, spray drying has effectively replaced other techniques, even in pharmaceutical studies, where the technique is primarily used for drying heat-sensitive compounds such as enzymes and pharmaceutical proteins without significantly losing activity, masking the taste of bitter active ingredients, enhancing the absorption of poorly soluble drugs due to the micronized particle sizes, improving the flow characteristics of microcapsules, coating the core materials, and formulating microcapillaries [73–75]. Spray drying is widely preferred over many other techniques because of its many benefits, which include its affordability, simplicity of operation, low water activity, production of fine particles, and suitability for transport and storage, can quickly treat materials, and offers some degree of control over the particle size distribution [19, 76]. It has been adopted across wide range of drug encapsulation, solubility enhancement, and taste masking; however, it has not been explored in designing HIV formulation despite the outstanding features and success.

In some studies, Priya Dharshini et al. [41] examined the *in vivo* pharmacokinetic analysis of dolutegravir-loaded spray-dried chitosan nanoparticles as milk admixture for pediatrics infected with HIV. Spray-dried chitosan nanoparticles coated with dolutegravir were created owing the drug's solubility problems. The produced nanoformulation's physicochemical characteristics were assessed, and its suitability for oral administration in combination with food or milk as an admixture for pediatric antiretroviral therapy was examined. In comparison to the pure drug, the nanoparticles showed an increase in *C*_max_. When compared to the AUC of animals given pure dolutegravir, the enhanced drug bioavailability in the nanoparticles given to mice further supported this phenomenon. Overall, they showed that chitosan-based nanoparticles could be a promising treatment adherence booster for pediatric HIV patients and be the perfect vehicle for the oral delivery of dolutegravir in combination with milk.

Spray drying technique has also been explored in designing the cyclodextrin-based pediatric anti-HIV formulations [77]. The authors produced a pediatric-friendly formulation of lopinavir, and ritonavir has given their poor palatability and poor solubility to achieve spray-dried drugs pediatric complexes. They showed enhanced dissolution profiles and improved taste of the spray-dried cyclodextrin complexes in comparison to pure drugs indicating the potential cyclodextrin in addressing the unmet need for the development of suitable pediatric formulations.

#### 2.2.2. Spray Chilling

**Figure 6:**
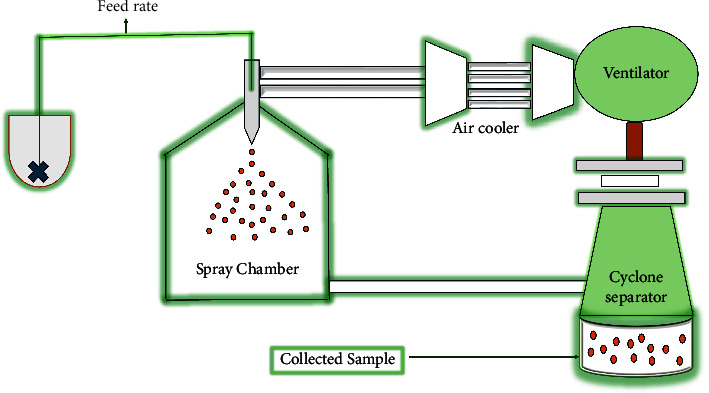
Graphical depiction of spray chilling encapsulation technique adapted from [78].

The spray chilling process (Figure 6), which shares many similarities with the spray drying method, is also known as spray cooling, spray congealing, or spray prilling. In contrast to the hot air produced by the spray drying method, this process requires mixing the core material and the carrier to create a slurry that is atomized with the chilled air [78, 79]. As opposed to spray drying, when the solvent evaporates, this procedure produces the particles by cooling and hardening the droplets of vegetable oil (32–42°C) employed for the hydrogenation and fractionation of the outer material. Other applications for the spray freezing technique include the encapsulation of heat-sensitive compounds and nonsoluble, frozen liquids [19, 24, 78]. Similar benefits to those of spray drying are provided by spray chilling, including the ability to mask unpleasant tastes or odors including HIV drugs for pediatrics, increase the solubility of poorly soluble core agents such as ARV drugs for potential sustained release, increase the stability of unstable ingredients, and decrease the hygroscopicity of individual ingredients. It does not require the use of organic solvents or high temperatures, is easy to operate, reasonably priced, and extremely scalable. Low entrapment efficiency and the release of the active ingredient upon storage are disadvantages of this process [46]. However, this technique can limit the challenging factors for children using HIV drugs including poor palatability of the drugs, the frequent administration, and use of the large pill due to the poor aqueous drug solubility and should be explored for this purpose.

#### 2.2.3. Extrusion

**Figure 7:**
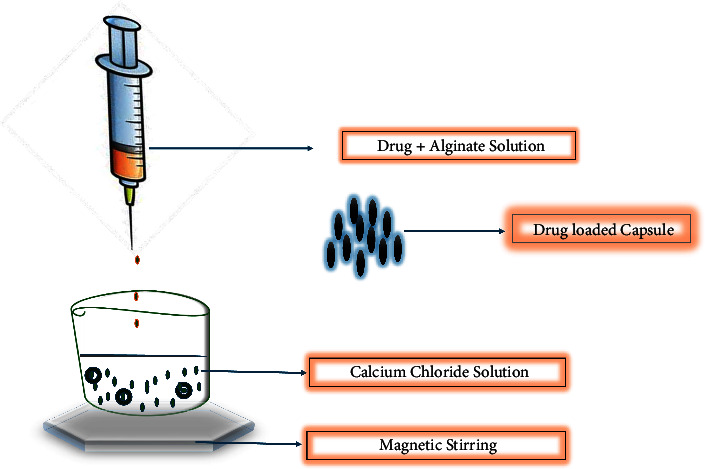
Schematic presentation of extrusion method adapted from [81].

Extrusion is one of the microencapsulation techniques which involves forcing a material to flow through an aperture with a different diameter at a predetermined rate under various conditions (high and low temperature, high and low moisture, and high and low speed) to produce various types of products based on consumer requirements or producer specifications [80]. Sodium alginate serves as the primary encapsulating agent in the extrusion process (Figure 7). The sodium alginate solution properly incorporates the active ingredient, which is immobilized by the robust polysaccharide gel created in the presence of the multivalent ion. The mixture is then subjected to a drop-wise extrusion into a hardening solution such as sodium chloride using a reduced calibre pipette or syringe. The extrusion method of processing has become an increasingly important manufacturing technique because it can operate continuously with high output and cheap production costs. Due to its low-moisture process, the extrusion method is environmentally beneficial because it no longer generates significant amounts of effluents, consequently, lowering the cost of water treatment and the level of environmental pollution [80]. This method has been heavily utilized mainly in the encapsulation of nutraceuticals, precarious flavors especially with the use of glassy carbohydrate as the coating agents but have not been explored towards HIV pediatric formulations despite the advancement and the potential [19]. This strategy can minimize the limitations associated with the treatment of HIV, including the large pill size and the poor solubility of the drugs which has contributed to therapeutic failure, in children. There are various extrusion technologies, and they are classified depending on the sort of extruder (a machine used for extrusion) being used, the extrusion condition, and other factors including the type of output being extruded. These methods of the extrusion technique are distinguished as follows:

1. Hot-melt
2. Melt injection
3. Centrifugal/coextrusion
4. Electrostatic and electrospinning
5. Particles from gas-saturated solution (PGSS)

The major advantage of this process is the improvement of the half-life of most of the compounds associated with oxidation problems. This is because the atmospheric gases disperse slowly through the hydrophilic glassy matrix and in the process creating an impermeable hurdle against oxygen. Although this method has been in existence, the formation of larger particles (500–1,000 mm) using this method has been its major challenges especially in the pharmaceutical and food industries where mouth feel is an important factor [19, 82]. The anti-HIV drug dolutegravir and its nanoparticles were incorporated into the buccal film that was manufactured deploying the produced polymer ink through research on the semisolid extrusion 3D printing technology [83]. An ideal viscosity for a smooth extrusion through the 3D printer's nozzle was achieved by processing the composite material consisting of polyvinyl alcohol and sodium alginate. The drug's disintegrating profile in the simulated salivary fluid was evaluated for buccal films both *in vitro* and *in vivo*, with an emphasis on examining the impact of polymer composition and printing conditions. According to the findings, the proposed polyvinyl alcohol-based polymer ink for pressure-based 3D printing provides a flexible method for producing mucoadhesive buccal films that can be tailored in terms of shape and drug loading.

#### 2.2.4. Fluidized Bed Coating

**Figure 8:**
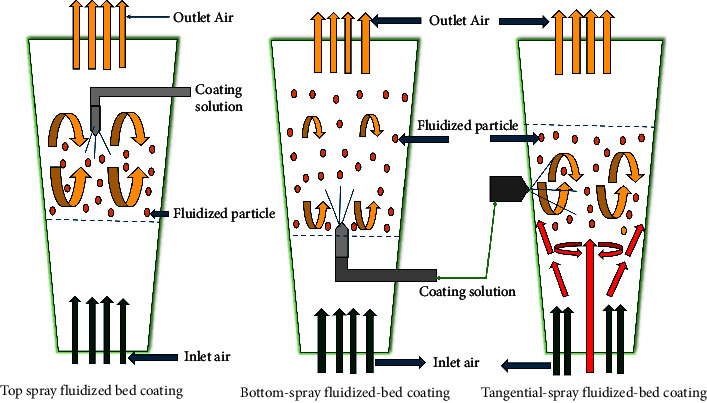
Schematic depiction of top, bottom, and tangential-spray fluidized-bed coating adapted from [71].

Fluidized bed coating is a type of spray coating microencapsulation technology which adopts suspending powder particles in a stream of air that is carefully regulated and persistently kept at a constant and specified temperature and humidity while continuously spraying with a coating material surface [84, 85]. Over time, the coating agents initiate the formation of a thin layer on the surface of the suspended particle. However, only coating materials with strong viscosity properties and thermal stability are taken into consideration in order to permit atomizing, pumping, and the creation of film over the particle's surface [84, 85]. The duration of the particles in the chamber where 5 to 50% of the coating is applied determines the coating of the particles using this technique. With the help of various coating agents, this process allows for the synthesis and manufacture of particles with diameters ranging from 50 to 500 microns [71]. The fluid-bed coating is made of different coaters (Figure 8) including the top spray, bottom spray, and tangential spray [24, 71]. With air on the fluid bed, the coating solution is sprayed downward in a countercurrent fashion in the top spray system, allowing porous or solid particles to migrate and transfer to the coating zone and subsequently become microencapsulated. The encapsulation tends to grow with little agglomeration or cluster formation because of the opposing flows of the covering material and particles [24, 71]. When compared to bottom spray and tangential spray, the fluid-bed top spray coater is widely employed in microencapsulation to manufacture microcapsules between 2 and 100 m with a high yield of the encapsulated particles. The Wurster technique, also known as the bottom spray, is employed to coat particles as small as 100 m. This method of coating consists of a coating chamber with a cribriform bottom plate and a cylindrical stainless-steel nozzle that sprays the coating material [71, 86]. The coating material microencapsulates the particles as they advance from the bottom to the top through the cribriform bottom plate and the nozzle zone, continuing until the appropriate thickness and weight are reached. The solvent is subsequently evaporated, and the microcapsules solidify and become rigid [71, 86]. The fluidized-bed coating processes use ethanolic solutions of synthetic polymers and mostly aqueous solutions such as gums, fats, or waxes to recoat already spray-dried microparticles for an improved half-life and protection. This method has the potential to produce controlled-release formulations more than any other coating technologies [24, 71, 86]. This approach can be explored in HIV pediatric formulations given its advantages such as the high rate of moisture removal, good thermal efficiency, low maintenance costs, and the flexibility to move items about the dryer with ease. However, high electrical power consumption, large pressure drops, agglomeration of the fine powder, and production of a nonuniform product have been its major challenges [87].

#### 2.2.5. Liposomal Encapsulation

**Figure 9:**
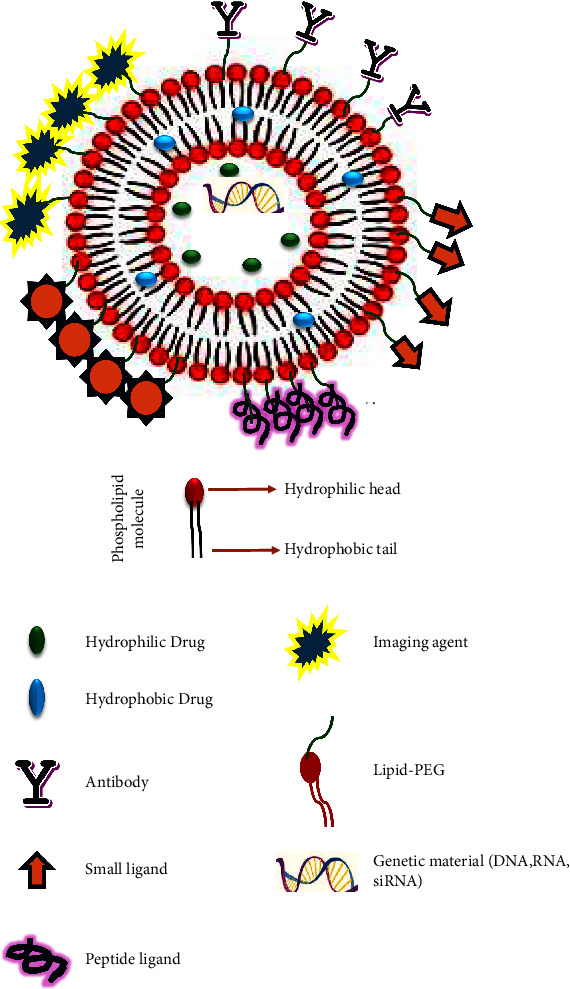
The depiction of functionalized liposomes adapted from [88].

Liposomes are the smallest and most spherical artificial vesicles made of either natural or synthetic phospholipids (Figure 9) [89]. Due to the many and varied benefits, it provides, such as the biocompatibility, biodegradability, low toxicity, tendency to encapsulate both hydrophilic and hydrophobic drugs, ability to target a specific site of action, function as a solubilizing agent, and ability to be modified to control their biological behavior due to their physicochemical and biophysical features, liposomes have received extensive research [19, 89–91]. Most industries have used liposomes for drug delivery, including the transfer of vitamins, hormones, enzymes, and flu shots into the body [89]. The United States Food and Drug Administration (FDA) and other regulatory bodies have recognized the manufacturing and clinical characteristics of liposomes, including batch-to-batch variability, formulation simplicity, scalability, and biocompatibility, as an acceptable formulation technique. Permeability, stability, surface activity, and affinity of liposomes might vary depending on their size and lipid makeup [92]. The diameter of liposomes can range from 25 nm to several microns, and they can be freeze-dried for storage. Phosphatidylcholine (PC), often known as lecithin, is the most widely employed phospholipid when making liposomes. It is composed of two hydrophobic fatty acid “tails” joined by a glycerol molecule, and a hydrophilic phosphate “head” [91]. The hydrophobic tails, which are made up of two long fatty acid chains, are attracted to water, whereas the hydrophilic head has a significant attraction for it [89, 93]. Due to their many advantages and applications, liposomes are now the most studied delivery technique and are used to encapsulate ARV drugs. They are well known for having minimal immunogenicity and cell selectivity and for using surface modifications to target drugs to virus-infected cells or organs, particularly HIV-infected cells [94]. Several novel liposomal approaches (immunoliposomes, long-circulating liposomes, long-circulating immunoliposomes, bioinspired liposomes, temperature, pH, and enzyme-sensitive liposomes) have all been investigated and established as a treatment strategy for different diseases and viruses including the HIV to specifically target the organ, cell, or tissue or the site of action [94, 95]. Although liposomes have been explored for pediatric formulations, however, liposomes have not fully been investigated for HIV pediatric formulations given the enormous potential especially they can be utilized to increase the solubility and may disguise the taste of ARV pediatric drugs. They can easily be modified to target the infected cells, achieve a liposome powder with reduced particle size to reduce frequent drug administration, difficulty in swallowing the large pills, and enhance therapeutic HIV pediatric treatment [94, 96].

The liposome delivery system has been investigated for HIV pediatric treatment, and in some cases, oral proliposomal dolutegravir powder for pediatrics has been developed [97]. The proliposomes demonstrated enhanced dissolution and improved the absorption rate compared to the pure drug indicating a potential application in HIV pediatric formulations.

A modified liposome (niosomes) for pediatric formulations has been prepared where different factors (physical and compositional factors) were evaluated to determine the features of the formulated niosomes using the high-pressure homogenizer [98]. The study depicted the importance of the microfluidization for the production and possible scale-up of anti-HIV niosomes with very small mean vesicular sizes for HIV pediatrics.

#### 2.2.6. Freeze Drying

**Figure 10:**
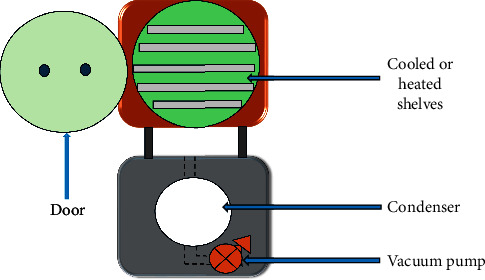
Schematic illustration of a freeze dryer adapted from [71].

Freeze drying, also known as lyophilization or cryodesiccation, is a microencapsulation process utilized largely in the dehydration of chemicals and materials, including scents and oils that are heat sensitive. Powders with high qualities have been produced by the use of freeze drying [99]. Given that the feed emulsion is frozen at a negative temperature, it is appropriate for bioactive substances that are delicate. The frozen fluid is then subjected to extremely low pressures, which cause the produced ice crystals to sublimate. Due to the use of a pump, which can create a vacuum, it is regarded as an expensive drying process. As a result, the lengthy (24–48 h) drying process involves a high-energy operation [99]. Using this technique, oil gets dissolved in the water and frozen between −90°C and −40°C with a decreased surrounding pressure and sufficient heat so as to allow the frozen water in the material to sublime. This technique (Figure 10) has been successfully adopted in the encapsulation of some water-soluble essences and aromas (oils) such as fish, flaxseed, walnuts, and olive oil. In addition to safeguarding heat-sensitive core components, freeze drying is straightforward and simple to use. Freeze-dried samples were found to have a higher level of oxidation resistance and a lower level of microencapsulation effectiveness possible [19, 71, 100]. When compared to other drying technologies, the main drawbacks are the high energy consumption, extended processing times, and high production costs, the porosity of the freeze-dried materials, which could expose the core material to the environment [71]. However, freeze-dried bioactive materials' porous architectures allow for a higher drug release [71].

The palatability of an innovative delivery mechanism utilizing a freeze drying in blister approach aimed at producing fast-dissolving tablets containing a fixed-dose combination of lopinavir and ritonavir was assessed [57]. The findings showed that freeze-dried rapid dissolving tablets with dual encapsulation and oral administration of lopinavir and ritonavir for pediatric HIV treatment in children when mixed with baby food could be a palatable delivery mechanism.

In another study involving freeze drying technique, an investigation by Lal et al. [58] was conducted on the production of fast-dissolving lopinavir and ritonavir tablets and was investigated through a study on the freeze drying blister approach. These tablets may be easily administered, even to newborns, by dispersing them in fluids and giving varied amounts of the dispersion according to their weight and age. For the distribution of ARV drugs, particularly to pediatric patients, specifically in low-resource environments, they described an appealing adjustable dosage form of the fast-dissolving tablets.

#### 2.2.7. Coacervation

**Figure 11:**
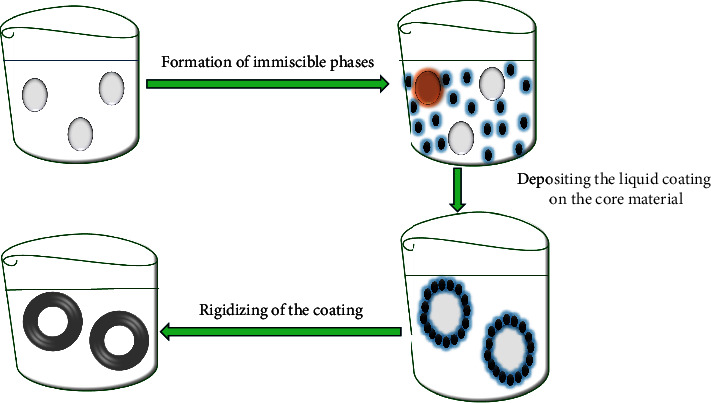
Illustration of microencapsulation by coacervation adapted from [101].

Coacervation process is one of the first and most popular methods for encapsulating different forms. Using this method (Figure 11), two biopolymers with opposing charges are brought together in a small pH range by electrostatic attraction, and at the conclusion of the procedure, the liquid phase displaces the rich phase, which is typically referred to as the coacervate [24, 101]. The method's propensity to entirely entrap the core material within the matrix is one of its key advantages. The simple coacervation and the complex coacervation are the two types of this procedure. In general, a single colloidal solute is added to the aqueous solution of a polymer during a simple coacervation process, whereas two colloids with opposing charges are added to a polymeric solution during a complex coacervation process. As a result, the complex coacervation process produces microcapsules through the ionic interactions of polymers with opposing charges, such as the positive charges of protein molecules and the anionic macromolecules of gelatin and Arabic gum [102]. The majority of the polymers utilized in the simple coacervation, however, are essentially nonsolvent or water-soluble polymers, including gelatin and gums, and are designed to create microcapsules by a hydrophobic contact with other polymers or proteins [101, 102]. When these two opposed charges balance each other out, the complex coacervate is created. With this technique, a liquid coating material phase is separated from a polymeric solution and coated as a homogeneous layer around suspended core particles [100]. When the system's total free energy is reduced, the coating material typically deposits on the surface of the core material and hardens by chemical or thermal cross-bonding to form solid capsules [24, 71, 101]. This process has been implemented on an industrial scale and can be adopted in HIV pediatric formulations. This is because it is very favorable over many other procedures in which it is scalable, uses few or no solvents, has a large payload, inexpensive, and reproducible, especially for the encapsulated drugs and even oils [102].

#### 2.2.8. *In Situ* Polymerization

**Figure 12:**
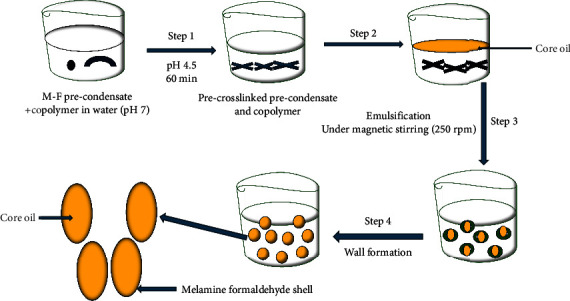
Illustration of in situ polymerization technique in the microencapsulation of essential oils adapted from [71].

This technology is regarded as the most popular microencapsulation process (Figure 12), which is used mostly to fabricate and produce functional fibres and microcapsules. When utilizing precipitants, a change in pH values, temperature, or solvent quality, the capsules in this process typically form on the particle's surface [60–62]. The absence of any reactants or reactive agents in the core material distinguishes this method for encapsulating from other polymerization processes. The main coating ingredients for the encapsulation utilizing this method include multifunctional monomers such as isocyanates and multifunctional acid chlorides, employed separately or in combination. These multifunctional monomers are typically dissolved in a liquid core material, dispersed in the aqueous phase of the dispersion agent, and then added to the mixture with a reactive multifunctional amine, which causes a fast polymerization at the interface to produce capsules [60–62]. A low molecular weight prepolymer will initially form; over time, the prepolymer will grow in size and accumulate on the surface of the scattered core material to create a solid capsule shell [61]. Depending on the polymerization monomer added to the reactor, many shell types are generated. The most common examples include the utilization of a urea shell made when isocyanate combines with amine, a polynylon or polyamide shell produced when acid chloride reacts with amine, and a polyurethane shell created when isocyanate reacts with a monomer that contains a hydroxyl group [19, 22, 61, 71]. A study involving *in situ* polymerization to develop a fixed-dose combination of lopinavir and ritonavir drugs in a children-friendly, flexible solid dosage form (granules) has been researched [63]. In rats given the commercial lopinavir/ritonavir pill, the results demonstrated improved bioavailability and markedly elevated lopinavir concentrations in examined tissues, particularly in HIV haven sites. Ultimately, the findings showed that the innovative *in situ* nanotechnology is an exciting option for producing flexible, tasty, and “heat” stable pediatric granules for fixed-dose combinations that can be utilized as sprinkles and sachets to treat HIV in children.

#### 2.2.9. Pan Coating

**Figure 13:**
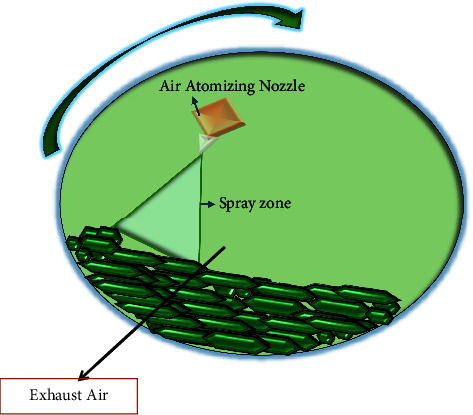
Schematic representation of pan coating adapted from [103].

Pan coating is one of the pharmaceutical industry's oldest and most popular spray coating process for producing coated particles or smaller-sized tablets (Figure 13). This method involves gently applying the coating substance throughout the coating process while the particles are being gently swirled in a pan or other devices. For efficient coating, solid particle sizes exceeding 600 m are typically regarded as necessary [103]. This method has been widely used in the formation of controlled-release beads and can also be utilized in the preparation of ARV formulations including pediatric beads. This technique involves coating the active chemicals onto spherical substrates such as nonpareil sugar seeds, followed by the application of protective coatings made of different polymers [16]. It is capable of enhancing the solubility of the core material, producing fine particles even smaller particle sizes; thus, a good shout-out for HIV pediatric formulations. In practice, the coating is applied to the desired solid core material in the coating pan as a solution or an atomized spray. Warm air is then passed over the coated materials to remove the coating solvent as the coatings are applied in the coating pans, and in some cases, the final solvent removal is done in the drying oven [16, 22, 103].

#### 2.2.10. Electrospraying

**Figure 14:**
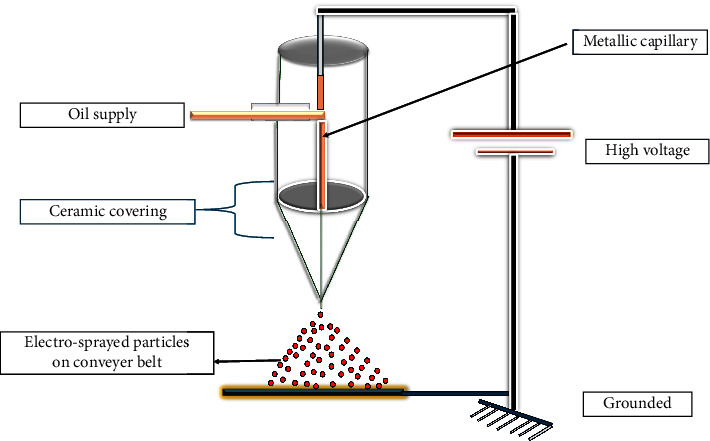
A schematic representation of electrospraying process through a single-nozzle system [104].

The electrospraying commonly known as electrohydrodynamic spraying (Figure 14) is a microencapsulation technique for atomizing liquids using electrical forces. Continuously flowing liquid is driven by an electric field into a dispersion of tiny, highly charged droplets by a capillary nozzle that is kept at a high electric potential [105]. In order to produce electrically charged polymer jets, the basic concepts of this approach involve using electrical shear to overcome the surface tension force of a pendant droplet of the biopolymer solution at the capillary nozzle [106, 105]. When an electric field is applied to a droplet, an electric charge is produced that competes with the particle's cohesive force. When it does, the surface tension is reduced and eventually nanoparticles are produced [107]. The size and morphology of the nanoparticles produced are affected by variables including the concentration of the polymer, shear viscosity, molecular weight of the polymer/solvent, and the electrospraying process including the electric potential, the electric difference, flow rate, and distance between the tip of the needle and the collector [104, 105, 107]. Electrospraying is popular in forming drug carriers for biomedical applications since it has few advantages over other traditional approaches. Since it is operated at room temperature, it can also be utilized to encapsulate delicate biomolecules and even living cells. Due to the potential absence of an external medium that would otherwise permit the disintegration or migration of water-soluble cargos, the encapsulation efficiency utilizing this technology is maximized [105]. This technique can reliably produce drug-loaded particles including ARV drug-loaded particles for children with a narrow distribution between 5 nm and 100 m in size. Hollow particles, porous microparticles, cell-shaped microparticles, and even multilayered microspheres might all be designed using this method [105, 108, 109].

## 3. Clinical Advances of Microparticulate Technologies and Microparticles in HIV Formulations

The pharmaceutical industry has successfully implemented the microencapsulation technology to tailor the delivery of drugs to certain organs and sites, mask the poor taste, improve drug stability, and control release patterns [110]. The release rates of microencapsulated APIs are precisely regulated at the intended areas, in contrast to standard drug delivery methods such as tablets, capsules, and syrups. Therapeutic efficacies are improved, and adverse effects are decreased due to their capacity to circumvent certain bodily areas [16, 111]. The conventional drug delivery method is vastly outmoded by the numerous applications of microencapsulated drug. Pursuant to the numerous benefits of these microencapsulation techniques, several pharmaceutical products containing microparticles are currently on the market (Table 3), and a number more are undergoing and had completed clinical trials to treat a variety of diseases, including HIV (Table 4) [110]. Multiple drugs belonging to distinct therapeutic groups have been encapsulated in microparticles and are presently undergoing thorough examination for potential clinical applications, especially with respect to diverse administration routes [110].

## 4. Conclusion and Future Prospectives

Several factors, including pill size and quantity, which is the main cause of swallowing difficulty, repeated administration of numerous ARVDs, many of which have poor solubility and severe side effects in children, unpalatability of the drug, which is one of the criteria for pediatric formulations, and this has promoted poor adherence and therapeutic failure among the children. Microencapsulation technology has been investigated across several fields, and many others are still being developed and discovered mostly in the pharmaceutical industries for the encapsulation of the APIs within a coating agent to produce different morphological microparticulates and nanoparticles of varying sizes with an improved pharmacological property required for an effective treatment. Although number of these technologies offer remarkable qualities and a unique way of manufacturing distinct types of microparticles of varying sizes, there has not been much focus on investigating these strategies in HIV pediatric formulations. Given that they are powerful in improving shelf life of the core material, enhancing the stability and solubility of the drugs, taste masking of the unpalatable drugs. For future HIV pediatric formulations, these technologies can be explored to improve palatability and taste masking properties of oral dosage form, investigated for use in the formulation of several HIV pediatric oral dosage forms including powders for reconstitution, multiparticulates, orodispersible or chewable tablets, micropellets, and other drug-resonant forms. They can also be applied for coencapsulation of the drugs, adopt in surface modification of the nano- or microparticle formulations to specifically target the site of action in addition. The 3D printing technology should also be investigated for HIV pediatric formulations. This is because 3D printing enables the manufacture of pharmaceutical dosage forms according to patient requirements, such as dose, release profile, color, texture, and size. Despite the tremendous success of these methods in producing microencapsulated commercially available products, there is still a long way to go before these technologies reach their full potential. To this effect, more studies must focus on the mechanisms underlying microencapsulation processes, as well as on the evaluation of the physicochemical properties of the individual drug and polymer, the stability of the encapsulated drugs, and the translation of bench-scale processes to industrial scales. By addressing these challenges, microencapsulation technology will evolve to an exciting new degree, enabling the realization of ever-more complex pharmaceutical drug systems.

## Figures and Tables

**Table 1:** Structures and names of some FDA-approved antiretroviral drugs [8].

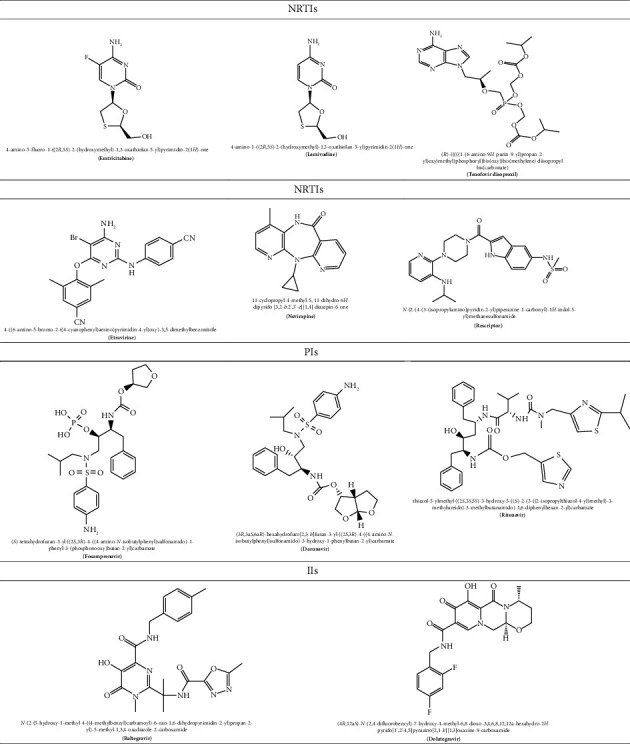

**Table 2:** List of some microencapsulated HIV drugs.

Drug (trade name and company)	Indication	Microencapsulation technology	Adult formulation	Pediatric formulation	Ref.
Lopinavir/ritonavir (Kaletra®, AbbVie)	HIV infections	Hot-melt extrusion	Melt-extruded solid dispersion	Oral solution	[35, 36]
Lopinavir/ritonavir (Cipla Ltd.)	HIV infections	Hot-melt extrusion	—	Oral pellets in capsules	[35]
Ritonavir (Norvir®, AbbVie)	HIV infection	Hot-melt extrusion	Film-coated tablets (100 mg)	Oral solution (80 mg/ml) Oral powder (100 mg packet)	[35]
Etravirine (Intelence®, Janssen-Cilag)	HIV infection	Spray drying	Tablets (100 or 200 mg)	Tablets (25 mg, 100 mg, and 200 mg)	[35]
Amprenavir (Agenerase®, GSK)	HIV infection	—	Soft gelatin capsules (50 and 150 mg)	Oral solution (15 mg/ml)	[35]
Tipranavir (Aptivus_, Boehringer Ingelheim)	HIV antiviral	—	Soft gelatin capsules (250 mg)	Oral solution (100 mg/mL)	[35]
Efavirenz/Sustiva®_oral solution/Bristol-Meyers Squibb		Lipid-based microemulsion	Hard gelatin capsules (50 mg, 100 mg, and 200 mg)	Oral solution (30 mg/ml)	[35]
Doxorubicin (DaunoXome®)	HIV-related Kaposi's sarcoma	Liposomes	Citrate liposome injection	—	[37]

**Table 3:** The demonstration of different novel microencapsulation techniques, core materials, drugs, and products in HIV treatment.

Microencapsulation technique	Loaded HIV drug	Synthesized formulation/products	Excipients	Administration route	Comments	Ref.
Spray drying	Tenofovir, dolutegravir, lamivudine, and efavirenz	Microspheres and nanoparticles	Soy extract, serum albumin, mannitol, calcium carbonate, starch, leucine, cyclodextrin, chitosan, and Eudragit S-100 sodium salt	Pulmonary, injection/vaccine, parenteral, oral, vaginal, intramuscular, or subcutaneous injectable	Scalable, simple and fast, fine particles, loss of samples, and possible agglomeration	[38–44]
Spray chilling		—	Starch, whey protein isolates, maltodextrins, chitosan, corn syrup, dextran, modified starch, cyclodextrins, lauric acid, oleic acid, gum arabic	Oral, pulmonary	Inexpensive, limited organic solvent, environmentally friendly, low or no heat, low encapsulation capacity, and possible degradation of the carrier	[19, 43, 45–47]
Extrusion	Zidovudine and stavudine	Microspheres, microcapsules, and microparticles	Gum sterculia, sodium alginate, chitosan cellulose: hydroxypropyl cellulose, hydroxypropyl methyl cellulose, ethyl cellulose, xanthan gum, starch, poly (lactic acid-co-glycolic acid) (PLGA) poly (caprolactone)	Nasal, oral	Easy scalability, solvent-free, economical, high-energy input, and high requirement of material properties	[48–51]
Fluidized bed coating	Emtricitabine and tenofovir	Film-coated tablets	Starch, dextran, sucrose, stearic acid, bees wax, phospholipids, and cellulose	Oral, pulmonary, vaginal, and skin	Uniform layer of coating materials on solid particles, controlled drug release, and poor control over air stream and air temperature	[43, 52, 53]
Liposomal encapsulation	Dolutegravir, efavirenz, tenofovir, and indinavir	Liposomes nanoformulations and buccal films	Lipids: wax, paraffin, diacylglycerols, lecithin, oils, and fats	Oral, buccal, transdermal, and vaginal	Coencapsulation, targeted release, biocompatible, and poor scalability	[19, 54–56]
Freeze drying	Lopinavir and ritonavir	Freeze-dried fast-dissolving tablets	Starch, maltodextrins, sucrose chitosan, corn syrup, dextran, modified starch, and cyclodextrins	Oral	Quality powders, straightforward, high-energy operation, and expensive	[19, 57, 58]
Coacervation	Stavudine	Gelatin liposomes	Starch, maltodextrins, sucrose chitosan, corn syrup, dextran, modified starch, and cyclodextrins	Oral	Little solvent, large payload, scalable, and reproducible	[19, 59]
*In situ* polymerization	Fixed-dose combination of lopinavir and ritonavir	Granules	Urea, melamine, formaldehyde, isocyanates, and acid chlorides	Oral	Polymerization in continuous phase and stable emulsion	[60–63]
Pan-coating	—		Ethyl cellulose, hydroxymethyl cellulose, lactose, and sodium carboxymethyl cellulose polyethylene powder	—	Low cost, controlled drug release, and difficult to control	[64, 65]
Electrospraying	—		Chitosan, lecithin, poly (lactide-coglycolide), whey protein isolate, soy protein isolate, ethyl cellulose, and methoxy citric pectin	—	High encapsulation efficiency, encapsulation of delicate biomolecule, and particle size between 5 and 100 nm	[66–68]

**Table 4:** Completed clinical studies exploring the contribution of liposomes [112, 113].

Condition	Treatment source	Enrolment	Phase	Sponsor	Clinical trial number
Severe AIDS-related Kaposi's sarcoma	Doxorubicin hydrochloride (liposomal)	—	Phase 3	Sequus Pharmaceuticals	NCT00002147
Kaposi's sarcoma in patients with AIDS	Comparison of liposomal doxorubicin used alone or in combination with bleomycin plus vincristine	120	Phase 2	National Institute of Allergy and Infectious Diseases (NIAID)	NCT00001059
AIDS-related Kaposi's sarcoma	Randomized, comparative trial of DOX-SL (stealth liposomal doxorubicin hydrochloride) versus bleomycin and vincristine	220	Phase 3	Sequus Pharmaceuticals	NCT00002105
AIDS-related Kaposi's sarcoma	Doxorubicin hydrochloride (liposomal) (DOX-SL)	—	Phase 3	Sequus pharmaceuticals	NCT00002319
AIDS	Safety and efficacy of amphotericin B lipid complex in the treatment of cryptococcal meningitis	—	NA	Liposome	NCT00002019
HIV infection	Microparticulate formulation of HIV-1 peptide vaccine		Phase II	National Institute of Allergy and Infectious Diseases	—
HIV infections	Microencapsulated DNA	—	Phase II	National Institute of Allergy and Infectious Diseases	—
HIV-associated Kaposi's sarcoma	A randomized phase III clinical trial of DaunoXome versus combination chemotherapy (drug: daunorubicin (liposomal); drug: bleomycin sulfate; drug: vincristine sulfate; drug: doxorubicin hydrochloride)	—	Phase 3	Nexstar Pharmaceuticals	NCT00002093

## Data Availability

This work is entirely dependent on previously published publications; hence, no data are generated or utilized in any way.

## Additional Points

*Statement*. The review was conceptualized from a component of my doctoral thesis titled “Investigation of microencapsulation techniques in the preformulation of selected antiretroviral drugs” which was submitted to the department of pharmaceutical sciences, the University of the Western Cape, South Africa [114]. Thus, the work was rewritten, transformed, proofread, and figures redrawn before being submitted for publication by the author.

## References

[1] Schlatter A. F., Deathe A. R., Vreeman R. C. (2016). The Need for Pediatric Formulations to Treat Children with HIV. *AIDS Research and Treatment*.

[2] Okafor N. I., Nkanga C. I., Waker R. B., Xavier S., Krause R. W. M. (2019). Encapsulation and physicochemical evaluation of efavirenz in liposomes. *Journal of pharmaceutical Investigation*.

[3] The Joint United Programme on HIV/AIDS (UNAIDS) (2019). *Global HIV and AIDS statistics-Fact sheet*.

[4] Pontali E., Feasi M., Toscanini F. (2001). Adherence to combination antiretroviral treatment in children. *HIV Clinical Trials*.

[5] Phelps B R, Rakhmanina N. (2011). Antiretroviral drugs in pediatric HIV-infected patients. *Pediatric Drugs*.

[6] Ramana L. N., Sharma S., Sethuraman S., Ranga U., Krishnan U. M. (2012). Investigation on the stability of saquinavir loaded liposomes: implication on stealth, release characteristics and cytotoxicity. *International Journal of Pharmaceutics*.

[7] Arage G., Tessema G. A., Kassa H. (2014). Adherence to antiretroviral therapy and its associated factors among children at south wollo zone hospitals, Northeast Ethiopia: a cross-sectional study. *BMC Public Health*.

[8] Michaud V., Bar-Magen T., Turgeon J., Flockhart D., Desta Z., Wainberg M. A. (2012). The dual role of pharmacogenetics in HIV treatment: mutations and polymorphisms regulating antiretroviral drug resistance and disposition. *Pharmacological Reviews*.

[9] Simon V., Ho D., Abdool Karim Q. (2006). HIV/AIDS epidemiology, pathogenesis, prevention, and treatment. *The Lancet*.

[10] Sharland M., Bryant P. A. (2009). Paediatric HIV infection. *Medicine*.

[11] Gupta U., Jain N. K. (2010). Non-polymeric nano-carriers in HIV/AIDS drug delivery and targeting. *Advanced Drug Delivery Reviews*.

[12] Schlatter A. F., Deathe A. R., Vreeman R. C. (2016). The need for pediatric formulations to treat children with HIV. *AIDS Research and Treatment*.

[13] Dubrocq G., Rakhmanina N., Phelps B. R. (2017). Challenges and opportunities in the development of hiv medications in pediatric patients. *Pediatric Drugs*.

[14] Stulzer H. K., Tagliari M. P., Parize A. L., Silva M. A. S., Laranjeira M. C. M. (2009). Evaluation of cross-linked chitosan microparticles containing acyclovir obtained by spray-drying. *Materials Science and Engineering: C*.

[15] Hoang Thi T. H., Morel S., Ayouni F., Flament M. P. (2012). Development and evaluation of taste-masked drug for paediatric medicines-Application to acetaminophen. *International Journal of Pharmaceutics*.

[16] Singh M.N., Hemant K. S. Y., Ram M., Shivakumar H. G. (2010). Microencapsulation: A promising technique for controlled drug delivery. *Research in Pharmaceutical Sciences*.

[17] Tshweu L., Katata L, Kalombo L (2014). Enhanced oral bioavailability of the antiretroviral efavirenz encapsulated in poly (epsilon-caprolactone) nanoparticles by a spray-drying method. *Nanomedicine*.

[18] Timilsena Y. P., Haque A., Adhikari B. (2020). Encapsulation in the Food Industry: A Brief Historical Overview to Recent Developments. *Food and Nutrition Sciences*.

[19] Poshadri A., Kuna A. (2010). Microencapsulation technology: a review. *Journal of Research Angrau*.

[20] Dubey R., Shami T., Rao K. B. (2009). Microencapsulation Technology and Applications. *Defence Science Journal*.

[21] Paulo F., Santos L. (2017). Design of experiments for microencapsulation applications: A review. *Materials Science and Engineering: C*.

[22] Agnihotri N., Mishra R., Goda C., Arora M. (2012). Microencapsulation–a novel approach in drug delivery: a review. *Indo Global Journal of Pharmaceutical Sciences*.

[23] Estevinho B. N., Rocha F., Santos L., Alves A. (2013). Microencapsulation with chitosan by spray drying for industry applications - A review. *Trends in Food Science and Technology*.

[24] Gouin S. (2004). Microencapsulation: industria appraisal of existing technologies and trends. *Trends in Food Science Technology*.

[25] Desai K. G. H., Park H. J. (2005). Recent developments in microencapsulation of food ingredients. *Drying Technology*.

[26] Singh M., Menra J., Soni M., Prasad D., Prasad D. N. (2016). Microencapsulation and its various aspects: a review. *International Journal of Advanced Research*.

[27] Arenas-Jal M., Suñé-Negre J. M., García-Montoya E. (2020). An overview of microencapsulation in the food industry: opportunities, challenges, and innovations. *European Food Research and Technology*.

[28] França D., Messa L. L., Souza C. F., Faez R. (2019). Nano and microencapsulated nutrients for enhanced efficiency fertilizer. *Polymers for Agri-Food Applications*.

[29] Kouam J., Songmene V., Balazinski M., Hendrick P. (2013). *Microencapsulation and its Uses on Food Science and Technology: A Review*.

[30] Chen Z., Fang Y., Zhang Z. (2007). Synthesis and assessment of attractiveness and mating disruption efficacy of sex pheromone microcapsules for the diamondback moth, plutella xylostella (L.). *Chinese Science Bulletin*.

[31] Tolescu C., Fierascu I., Neamtu C., Anton I., Fierascu C. (2014). Microencapsulated fertilizers for plant nutrition improvement. *Journal of the Serbian Chemical Society*.

[32] Obeidat W. (2009). Recent patents review in microencapsulation of pharmaceuticals using the emulsion solvent removal methods. *Recent Patents on Drug Delivery and Formulation*.

[33] Walsh J., Ranmal S. R., Ernest T. B., Liu F. (2018). Patient acceptability, safety and access: A balancing act for selecting age-appropriate oral dosage forms for paediatric and geriatric populations. *International Journal of Pharmaceutics*.

[34] Penazzato M., Townsend C. L., Rakhmanina N. (2019). Prioritising the most needed paediatric antiretroviral formulations: the PADO4 list. *The Lancet HIV*.

[35] Salunke S., O’Brien F., Cheng Thiam Tan D. (2022). Oral drug delivery strategies for development of poorly water soluble drugs in paediatric patient population. *Advanced Drug Delivery Reviews*.

[36] Kaletra (2003). *Prescribing Information*.

[37] Nisini R., Poerio N., Mariotti S., De Santis F., Fraziano M. (2018). The multirole of liposomes in therapy and prevention of infectious diseases. *Frontiers in Immunology*.

[38] Serrano D. R., Hernández L., Fleire L. (2013). Hemolytic and pharmacokinetic studies of liposomal and particulate amphotericin B formulations. *International Journal of Pharmaceutics*.

[39] Sarrate R., Ticó J. R., Miñarro M. (2015). Modification of the morphology and particle size of pharmaceutical excipients by spray drying technique. *Powder Technology*.

[40] Narayanan V. H. B., Lewandowski A., Durai R., Gonciarz W., Wawrzyniak P., Brzezinski M. (2022). Spray-dried tenofovir alafenamide-chitosan nanoparticles loaded oleogels as a long-acting injectable depot system of anti-HIV drug. *International Journal of Biological Macromolecules*.

[41] Priya Dharshini K., Ramya Devi D., Banudevi S., Vedha V. H. (2022). In-vivo pharmacokinetic studies of Dolutegravir loaded spray dried chitosan nanoparticles as milk admixture for paediatrics infected with HIV. *Scientific Reports*.

[42] Zhang T., Zhang C., Agrahari V., Murowchick J. B., Oyler N. A., Youan B. B. C. (2013). Spray drying tenofovir loaded mucoadhesive and pH-sensitive microspheres intended for HIV prevention. *Antiviral Research*.

[43] Jeyakumari A., Zynudheen A. A., Parvathy U. (2016). Microencapsulation of bioactive food ingredients and controlled release-a review. *MOJ Food Processing and Technology*.

[44] Panizzon G. P., Bueno F. G., Ueda-nakamura T., Nakamura C. V., Dias Filho B., Filho D. (2014). Preparation of spray-dried soy isoflavone-loaded gelatin microspheres for enhancement of dissolution: formulation, characterization and in vitro evaluation. *Pharmaceutics*.

[45] Sartori T., Consoli L., Hubinger M. D., Menegalli F. C. (2015). Ascorbic acid microencapsulation by spray chilling: Production and characterization. *Lwt Food Science and Technology*.

[46] Okuro P. K., Eustáquio de Matos F., Favaro-Trindade C. S. (2013). Technological challenges for spray chilling encapsulation of functional food ingredients. *Food Technology and Biotechnology*.

[47] Deshpande D., Blanchard J, Srinivasan S (2002). Aerosolization of lipoplexes using AERx® pulmonary delivery system. *Journal of Pharmaceutical Sciences*.

[48] Panda S., Pattnaik S., Maharana L., Mahapatra A. K. (2014). Formulation and evaluation of zidovudine loaded biodegradable microcapsules employing sterculia gum by emulsification-internal gelation technique. *Latin of American Journal of Pharmacy*.

[49] Dey S., Pramanik S., Malgope A. (2011). Formulation and Optimization of Sustained Release Stavudine Microspheres Using Response Surface Methodology. *ISRN Pharmaceutics*.

[50] Ren Y., Mei L., Zhou L., Guo G. (2019). Recent perspectives in hot melt extrusion-based polymeric formulations for drug delivery: applications and innovations. *Journal of Pharmaceutical Sciences*.

[51] Dalpiaz A., Fogagnolo M., Ferraro L. (2015). Nasal chitosan microparticles target a zidovudine prodrug to brain HIV sanctuaries. *Antiviral Research*.

[52] Srilatha U., Krishna R. M., Reddy V. D., Reddy Devireddy S. (2015). Formulation and evaluation of emtricitabine and tenofovir disoproxil fumarate film coated tablets. *Internation Journal of Research in Pharmacy and Chenistry*.

[53] Choudhury N., Meghwal M., Das K. (2021). Microencapsulation: An overview on concepts, methods, properties and applications in foods. *Food Frontiers*.

[54] Faria M. J., Machado R., Ribeiro A. (2019). Rational development of liposomal hydrogels: A strategy for topical vaginal antiretroviral drug delivery in the context of HIV prevention. *Pharmaceutics*.

[55] Dubey V., Mishra D., Nahar M., Jain V., Jain N. K. (2010). Enhanced transdermal delivery of an anti-HIV agent via ethanolic liposomes. *Nanomedicine Nanotechnology, Biology and Medicine*.

[56] Okafor N. I., Ngoepe M., Noundou X. S., Maçedo Krause R. W. (2019). Nano-enabled liposomal mucoadhesive films for enhanced efavirenz buccal drug delivery. *Journal of Drug Delivery Science and Technology*.

[57] Pittman D. W., Brantly A. M., Drobonick A. L. (2018). The palatability of lopinavir and ritonavir delivered by an innovative freeze-dried fast-dissolving tablet formulation. *AIDS Research and Treatment*.

[58] Lal M., Lai M., Estrada M., Zhu C. (2017). Developing a flexible pediatric dosage form for antiretroviral therapy: a fast-dissolving tablet. *Journal of Pharmaceutical Sciences*.

[59] Nayak D., Boxi A., Ashe S., Thathapudi N. C., Nayak B. (2017). Stavudine loaded gelatin liposomes for HIV therapy: Preparation, characterization and in vitro cytotoxic evaluation. *Materials Science and Engineering C*.

[60] Hwang J. S., Kim J. N., Wee Y. J. (2006). Preparation and characterization of melamine-formaldehyde resin microcapsules containing fragrant oil. *Biotechnology and Bioprocess Engineering*.

[61] Jyothi N. V. N., Prasanna P. M., Sakarkar S. N., Prabha K. S., Ramaiah P. S., Srawan G. Y. (2010). Microencapsulation techniques, factors influencing encapsulation efficiency. *Journal of Microencapsulation*.

[62] Nguon O., Lagugné-Labarthet F., Brandys F. A., Li J., Gillies E. R. (2018). Microencapsulation by in situ polymerization of amino resins. *Polymer Reviews*.

[63] Pham K., Li D., Guo S., Penzak S., Dong X. (2016). Development and in vivo evaluation of child-friendly lopinavir/ritonavir pediatric granules utilizing novel in situ self-assembly nanoparticles. *Journal of Controlled Release*.

[64] Bobde M., Ghade P., Kale P., Shahi S. (2022). Microencapsulation and its various aspects: a review. *International Journal of Advanced Research*.

[65] Yang Y., Li W., Yu D. G., Wang G., Williams G. R., Zhang Z. (2019). Tunable drug release from nanofibers coated with blank cellulose acetate layers fabricated using tri-axial electrospinning. *Carbohydrate Polymers*.

[66] Coghetto C. C., Brinques G. B., Siqueira N. M., Pletsch J., Soares R. M. D., Ayub M. A. Z. (2016). Electrospraying microencapsulation of Lactobacillus plantarum enhances cell viability under refrigeration storage and simulated gastric and intestinal fluids. *Journal of Functional Foods*.

[67] Wang P., Ding M., Zhang T. (2022). Electrospraying technique and its recent application advances for biological macromolecule encapsulation of food bioactive substances. *Food Reviews International*.

[68] Feng K., Huangfu L., Liu C. (2023). Electrospinning and electrospraying: emerging techniques for probiotic stabilization and application. *Polymers*.

[69] Yasuji T., Takeuchi H., Kawashima Y. (2008). Particle design of poorly water-soluble drug substances using supercritical fluid technologies. *Advanced Drug Delivery Reviews*.

[70] Abuzar S., Hyun S. M., Kim J. H. (2018). Enhancing the solubility and bioavailability of poorly water-soluble drugs using supercritical antisolvent (SAS) process. *International Journal of Pharmaceutics*.

[71] Bakry A. M., Abbas S., Ali B. (2015). Microencapsulation of oils: a comprehensive review of benefits, techniques, and applications. *Comprehensive Reviews in Food Science and Food Safety*.

[72] Phisut N. (2012). Spray drying technique of fruit juice powder: some factors influencing the properties. *International Food Research Journal*.

[73] Eun J. B., Maruf A., Das P. R., Nam S. H. (2020). A review of encapsulation of carotenoids using spray drying and freeze drying. *Critical Reviews in Food Science and Nutrition*.

[74] Psimadas D., Georgoulias P., Valotassiou V, Loudos G (2012). Molecular nanomedicine towards cancer: 111in-labeled nanoparticles. *Journal of Pharmaceutical Sciences*.

[75] Seremeta K. P., Tur M. I. R., Pérez S. M. (2014). Spray-dried didanosine-loaded polymeric particles for enhanced oral bioavailability. *Colloids and Surfaces B: Biointerfaces*.

[76] Ré M. I. (2006). Formulating drug delivery systems by spray drying. *Drying. Technology*.

[77] Ivone R., Fernando A., DeBoef B., Meenach S. A., Shen J. (2021). Development of spray-dried cyclodextrin-based pediatric anti-HIV formulations. *AAPS PharmSciTech*.

[78] Oxley J. D. (2012). *Spray cooling and spray chilling for food ingredient and nutraceutical encapsulation*.

[79] Risch S. J., and Reineccius R. (1995). Encapsulation: overview of uses and techniques. *Encapsulation and Controlled Release of Food Ingredients*.

[80] Bamidele O. P., Emmambux M. N. (2021). Encapsulation of bioactive compounds by ‘extrusion’ technologies: a review. *Critical Reviews in Food Science and Nutrition*.

[81] Liliana S. C., Vladimir V. C. (2013). Probiotic encapsulation. *African Journal of Microbiology Research*.

[82] Silva P. T. D., Fries L. L. M., Menezes C. R. D. (2014). Microencapsulation: concepts, mechanisms, methods and some applications in food technology. *Ciência Rural*.

[83] Krishnan P. D., Durai R. D., Veluri S., B Narayanan V. H. (2024). Semisolid extrusion 3D printing of dolutegravir-chitosan nanoparticles laden polymeric buccal films: personalized solution for pediatric treatment. *Biomedical Materials*.

[84] Dewettinck K., Huyghebaert A. (1999). Fluidized bed coating in food technology. *Trends in Food Science and Technology*.

[85] Teunou E., Poncelet D. (2005). Fluid-bed coating. *Encapsulated and powdered food*.

[86] Kaushik P., Dowling K., Barrow C. J., Adhikari B. (2015). Microencapsulation of omega-3 fatty acids: A review of microencapsulation and characterization methods. *Journal of Functional Foods*.

[87] Law C., Mujumdar A. (2014). Fluidized bed dryers. *Handbook of industrial drying*.

[88] Riaz M. K., Riaz M. A., Zhang X. (2018). Surface functionalization and targeting strategies of liposomes in solid tumor therapy: A review. *International Journal of Molecular Sciences*.

[89] Akbarzadeh A., Rezaei-Sadabady R., Davaran S. (2013). Liposome: Classification, preparation, and applications. *Nanoscale Research. Letters*.

[90] Sahoo S. K., Labhasetwar V. (2003). Nanotech approaches to drug delivery and imaging. *Drug Discovery Today*.

[91] Mansoori M., Agrawal S., Jawade S., K M. (2012). A Review on liposome. *International Journal of Advanced Research in Pharmaceutics and Bioscience*.

[92] Nkanga C. I., Bapolisi A. M., Okafor N. I., Werner Krause R. (2019). General perception of liposomes: formation, manufacturing and applications. *IntechOpen*.

[93] Sercombe L., Veerati T., Moheimani F., Wu S. Y., Sood A. K., Hua S. (2015). Advances and challenges of liposome assisted drug delivery. *Frontiers in Pharmacology*.

[94] Chopra S., Venkatesan N., Betageri G. V. (2013). Liposomes as nanocarriers for anti-HIV therapy. *Drug Delivery and Translational Research*.

[95] Okafor N. I., Nkanga C. I., Walker R. B., Noundou X. S., Krause R. W. M. (2019). Encapsulation and physicochemical evaluation of efavirenz in liposomes. *Journal of Pharmaceutical Investigation*.

[96] Sosnik A., Augustine R. (2016). Challenges in oral drug delivery of antiretrovirals and the innovative strategies to overcome them. *Advanced Drug Delivery Reviews*.

[97] Shaik N. B., Shakelli D., Lakshmi PK L., Rao VV B. (2020). Formulation and evaluation of dolutegravir proliposomal powder for pediatric HIV patients. *International Journal of Pharmaceutical Investigation*.

[98] Zidan A. S., Rahman Z., Khan M. A. (2011). Product and process understanding of a novel pediatric anti-HIV tenofovir niosomes with a high-pressure homogenizer. *European Journal of Pharmaceutical Sciences*.

[99] Pudziuvelyte L., Marksa M., Sosnowska K., Winnicka K., Morkuniene R., Bernatoniene J. (2020). Freeze-drying technique for microencapsulation of elsholtzia ciliata Ethanolic extract using different coating materials. *Molecules*.

[100] Velasco J., Holgado F., Dobarganes C., Márquez-Ruiz G. (2009). Antioxidant activity of added phenolic compounds in freeze-dried microencapsulated sunflower oil. *Journal of the American Oil Chemists’ Society*.

[101] Kashif I., Khan A., Sun D. (2019). Phase change materials , their synthesis and application in textiles—a review. *The Journal of Textile Institute*.

[102] Xiao Z., Liu W., Zhu G., Zhou R., Niu Y. (2014). Production and characterization of multinuclear microcapsules encapsulating lavender oil by complex coacervation. *Flavour and Fragrance Journal*.

[103] Bansode S. S., Banarjee S. K., Gaikwad D. D., Jadhav S. L., Thorat R. M. (2010). Microencapsulation: a review. *International Journal of Pharmaceutical Sciences Review and Research*.

[104] Khan M. K. I., Nazir A., Maan A. A., Maan A. A. (2016). Electrospraying: a novel technique for efficient coating of foods. *Food Engineering Reviews*.

[105] Taheri A., Jafari S. M. (2019). 18 - Nanostructures of gums for encapsulation of food ingredients. *Biopolymer Nanostructures for Food Encapsulation Purposes*.

[106] Augustin M. A., Oliver C. M. (2012). An overview of the development and applications of nanoscale materials in the food industry. *Nanotechnology in the Food, Beverage and Nutraceutical Industries’*.

[107] Tapia-hernández J. A., Rodríguez-félix F. (2017). Nanocapsule formation by electrospraying. *Nanoencapsulation Technologies for the Food and Nutraceutical Industries*.

[108] Wang J., Jansen J. A., Yang F. (2019). Electrospraying: possibilities and challenges of engineering carriers for biomedical applications—a mini review. *Frontiers in Chemistry*.

[109] Raval D., Kabariya J., Hazra T., Ramani V. (2019). A review on electrospraying technique for encapsulation of nutraceuticals. *International Journal of Chemical Studies*.

[110] Yan C., Kim S. R. (2024). Microencapsulation for pharmaceutical applications: a review. *ACS Applied Bio Materials*.

[111] Adepu S., Ramakrishna S. (2021). Controlled drug delivery systems: current status and future directions. *Molecules*.

[112] Saeedi M., Mehranfar F., Omidi S., Ehsani F. Z., Pajand O. (2020). Biological aspects and clinical applications of nanoparticles on treatment and prophylaxis of HIV. *Iranian Journal of Medical Microbiology*.

[113] Lu Y. (2012). Microencapsulation: methods and pharmaceutical applications. *Encyclopedia of Pharmaceutical Science and Technology*.

[114] Okafor N. I. (2022). *Investigation of Suitable Microencapsulation Techniques in the Preformulation of Selected Antiretroviral Drugs*.

